# A Review on Soils Treated with Biopolymers Based on Unsaturated Soil Theory

**DOI:** 10.3390/polym15224431

**Published:** 2023-11-16

**Authors:** Junran Zhang, Jiahao Liu

**Affiliations:** Henan Province Key Laboratory of Geomechanics and Structural Engineering, North China University of Water Resources and Electric Power, Zhengzhou 450046, China

**Keywords:** biopolymer-treated soil, strength, water retention characteristics, permeability, durability

## Abstract

Adding different materials to soil can improve its engineering properties, but traditional materials such as cement, lime, fly ash, etc., have caused pollution to the environment. Recently, biopolymers have shown many advantages, such as economy and environmental protection, which make them applicable to geotechnical engineering. This study summarizes the effects of biopolymers on soil’s engineering properties and the main directions of current research. Firstly, the advantages and disadvantages of a variety of widely used biopolymer materials and their effects on the specific engineering characteristics of soil (i.e., water retention characteristics, strength characteristics, permeability characteristics, microstructure) are introduced, as well as the source, viscosity, pH, and cost of these biopolymers. Then, based on the theory of unsaturated soil, the current research progress on the water retention characteristics of improved soil is summarized. The key factors affecting the strength of biopolymer-treated soil are introduced. Due to the actual environmental conditions, such as rainfall, the permeability and durability of biopolymer-treated soil are also worthy of attention. In summary, it is necessary to study the variation laws of the engineering properties of biopolymer-treated soil in the full suction range, and to predict such laws reasonably. The relevant results are conducive to the safer and more scientific application of biopolymers in geotechnical engineering practice.

## 1. Introduction

Soil and water loss is a serious environmental problem that aggravates land desertification and is accompanied by a series of natural disasters, which pose a serious threat to people’s lives and property. During construction projects, the construction can only be carried out when the local foundation soil exhibits suitable geotechnical properties. If the engineering properties of the foundation soil are poor, it is necessary to use a variety of techniques to improve its engineering properties, such as foundation soil compaction, gravel concrete columns, phased construction, and the use of cement and other binders to improve the properties of the soil (strength, erosion resistance, water-holding characteristics, etc.) [1]. Among them, there are many kinds of materials that can be used as adhesives, such as cement mortar, blast furnace slag [2], and fly ash [3]. However, due to the poor environmental protection performance of these materials, it is easy for them to cause many adverse effects on the environment. Therefore, it is necessary to use green materials that are conducive to ecological and environmental restoration.

Biopolymers are environmentally friendly materials, so people have begun to explore the application of biopolymers in geotechnical engineering in recent years. At present, there are many kinds of polymers that are used in geotechnical engineering, such as guar gum, gellan gum, xanthan gum, agar glue, and chitosan [4]. Although the use of biopolymers to improve soil quality can meet the requirements of environmental protection while ensuring the effectiveness of the project, it still needs to be further explored and verified in terms of site applicability [4], durability [5], and economic feasibility [1]. Considering the dry climate in many areas, the surface soil, foundation, and geotechnical structures above the groundwater level are in an unsaturated state, and the capillary action and dry–wet circulation are very strong. In the process of mutual transformation between unsaturated and saturated states, the water retention characteristics, strength characteristics, and permeability characteristics of biopolymer-treated soil will inevitably change. Therefore, it is difficult to accurately analyze and calculate when analyzing and designing important infrastructure (such as building foundations, dams, highways, slopes, tunnels, and waste protection facilities), often causing serious engineering accidents and geological disasters. For example, the shrinkage of soil caused by evaporation leads to cracks in the foundations of buildings, roads, and bridges; after rainfall, ditch slopes are unstable and destroyed, and the road surface is subsided and collapsed. Therefore, the change law of the engineering properties of biopolymer-treated soil in the range of total suction, along with its internal mechanisms and prediction methods, is very important and can provide a scientific basis for the safer and more scientific application of biopolymers in geotechnical engineering.

Laboratory experimental research on the engineering properties of biopolymer-treated soils is at a relatively mature stage. Based on the current research results, and combined with the actual engineering situation, it is an important direction and challenge for future research to extend biopolymer-treated soils to engineering field practice through more advanced test methods and technical means based on the unsaturated soil theory.

## 2. Biopolymers Commonly Used in Geotechnical Engineering

### 2.1. AGAR

AGAR is a polysaccharide, and most agarose is derived from plant extracts. The main property of AGAR gel is the ability to form a reversible gel by cooling an aqueous solution without the need for other chemical reagents. When agarose binds to water molecules, the water molecules will settle into the double-helix structure of the agarose molecules, contributing to the overall stability of the hydrogel [6]. In addition, due to its shear rheological properties, it has been widely used to improve soil strength and reduce environmental pollution [4]. AGAR is a typical thermogelated biopolymer. Due to its hydrogen-bonding properties, AGAR can be used to improve viscous and sandy soils [7,8] and to improve the strength and durability of soil. Hydrogen bonding between biopolymers and soil particles forms biopolymer–soil matrices, and this effect can last a long time. As for AGAR, time is not an important treatment consideration, while water content is a critical strengthening parameter for soil treatment [8].

### 2.2. Gellan Gum

Gellan gum is an anionic polysaccharide produced by microbial fermentation. It has different characteristics in different environmental states, mainly affected by temperature, water environment, and PH value, among which temperature is a key factor. Gellan gum presents a double-helix structure at low temperatures and transforms into a single-helix structure at high temperatures [9]. In geotechnical engineering, gellan gum is mostly used to treat non-cohesive soil. When gellan gum is combined with soil particles at high temperatures, a solid hydrogel can be formed to improve the strength of the soil, block the pores between soil particles, and improve the water retention performance of the soil. In addition, gellan gum has higher durability than other kinds of biopolymers, and it still has a good improvement effect under multiple dry and wet cycles [5]. However, the high-temperature environment required for the use of gellan gum is difficult to achieve in practical engineering, limiting its range of use in engineering.

### 2.3. Guar Gum

Guar gum is a neutral polysaccharide derived from the seeds of the guar bean, a leguminous plant. Guar gum has a high viscosity at low concentrations and can hydrate rapidly in cold water. The hydration time is an important factor affecting the effect of guar gum. If the hydration process takes a long time, the viscosity of the guar gum will decrease [10]. Many studies have shown that the hydration time of guar gum should be controlled within two hours to achieve a better effect. Guar gum is often used in geotechnical engineering to improve the water retention properties of soils [11].

### 2.4. Xanthan Gum

Xanthan gum is also a polysaccharide biopolymer derived from bacterial fermentation. The solution viscosity of xanthan gum increases linearly with the increase in xanthan gum content and exhibits high stability over a wide range of temperature, pH, and electrolyte concentration [12]. Because of its excellent stability, xanthan gum is often used in the food industry and the petroleum industry [12]. In addition, xanthan gum is easy to obtain, simple to process, and cost-effective, which is why it mainly used in geotechnical engineering to improve soil’s strength and water retention characteristics, and it has been applied in the engineering field [13].

### 2.5. Chitosan

Chitosan is a linear polysaccharide that is mainly derived from crustaceans and insects. This kind of polysaccharide has a similar molecular structure to human tissue and does not cause an immune response, so it is often used to produce thickeners and stabilizers for food and biological materials. In addition, chitosan is also a biodegradable material that is used in the production of environmentally friendly pesticides and crop fertilizers [14,15]. In geotechnical engineering, chitosan is used as a flocculant to treat pollutants in groundwater and remove harmful ions from groundwater [16,17]. In addition, the cationic charge of chitosan combines with the anionic charge in the soil particles to form sediment, which has a pore-blocking effect and can significantly reduce the hydraulic conductivity of the soil [18].

### 2.6. Starch

Starch, one of the most common natural biopolymers, is abundant in a variety of crops, such as corn, rice, wheat, potato, and cassava, and is composed of a variety of monosaccharide molecules [19]. Starch is widely used in food, plastics, cosmetics, pharmaceuticals, and other industrial fields, and it is commonly used as a thickener, a stabilizer, and a binder [20,21]. In geotechnical engineering, starch is mostly used to improve the mechanical properties of soil, including shear strength and elastic modulus [22]. The cementation of a starch solution and soil particles can improve the erosion resistance of the soil [23].

According to the chemical characteristics and use conditions of different biopolymers, the types of polymers should be selected reasonably to achieve better improvement results. Therefore, the performance of commonly used biopolymers in improving soils’ engineering characteristics needs to be studied more carefully to make a reasonable choice. Biopolymers that are commonly used in geotechnical engineering are shown in Table 1.

## 3. Water Retention Properties of Biopolymer-Treated Soils

**Figure 1:**
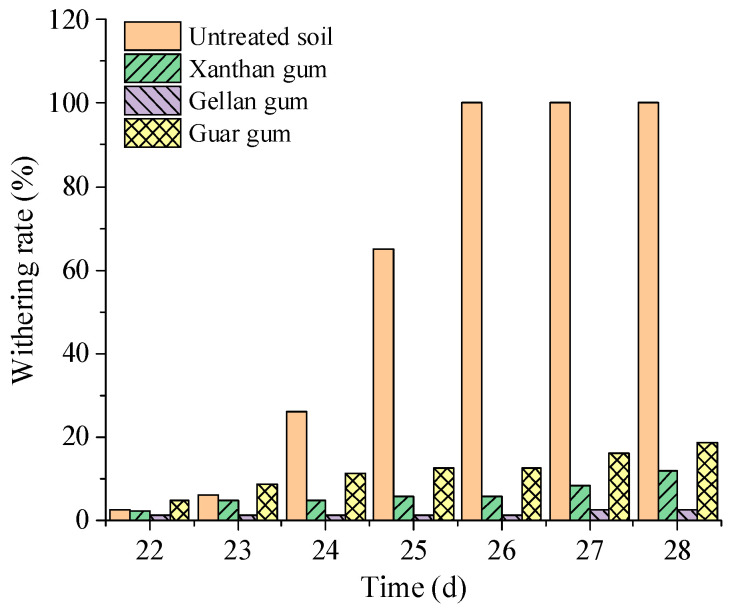
Vegetation wilting rate [11].

**Figure 2:**
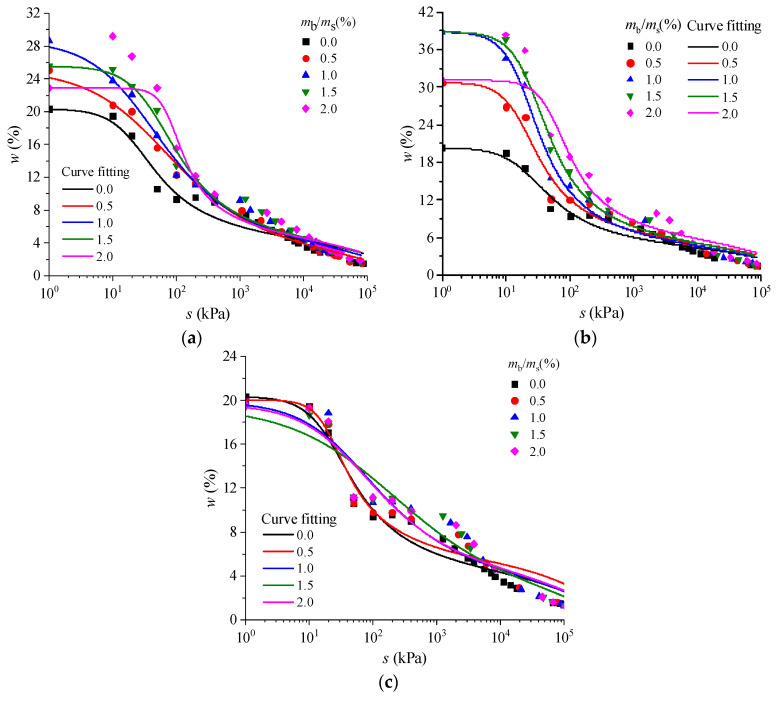
Soil–water characteristic curves of silty soil treated with three biopolymers: (**a**) xanthan gum, (**b**) gellan gum, and (**c**) guar gum [11].

The addition of biopolymers can significantly improve the water retention capacity of soil. Many studies have shown that the addition of xanthan gum significantly enhances the water retention characteristics of soil [24], which play an important role in retaining soil water, improving material moisture absorption performance, and increasing crop yields [25]. Biopolymers can be effectively used for the improvement of non-cohesive soil to enhance its water retention capacity, thus creating conditions for soil and water conservation and vegetation growth [11]. As shown in Figure 1, after stopping watering, the wilt rate of the biopolymer-treated silt was significantly lower than that of the untreated silt, and the vegetation blight rate of gellan-gum-treated silt was the lowest [11]. This indicates that gellan gum can effectively maintain soil moisture content and reduce the blight rate of vegetation. Figure 2 shows the soil–water characteristic curves of silty soils treated with xanthan gum, gellan gum, and guar gum [11], proving that gellan-gum-treated silt has better water retention characteristics than silts treated with the other two biopolymers-treated.

Compared with plain soil, the moisture content of xanthan-gum-treated sandy soil is higher after saturation. Chang et al. [4] proved that water content is the key factor affecting the soil strengthening of thermal gel biopolymers such as gellan gum and AGAR gum. Rainfall and evaporation are common phenomena in nature, and soil modified by xanthan gum can absorb a large amount of water in the rainfall season and has a higher water-holding capacity in the dry season [26], which is more conducive to the growth of vegetation [11].

As for unsaturated soil, the key to the experimental study of the water retention characteristics of biopolymer-treated soil is the control of the water content and the determination of the corresponding suction value. By controlling the moisture content, the suction value in a fixed range can be obtained, and then a complete soil–water characteristic curve can be fitted. The soil–water characteristic curve is of great significance to the study of biopolymer-treated soil based on unsaturated soil theory. Soil–water characteristic curves can be used to calculate many characteristics of unsaturated soil, including the permeability coefficient [27], shear strength [28], and volume strain, which are important parameters to determine the water retention capacity of unsaturated soil.

### 3.1. Review of Test Methods for the Water Retention Properties of Biopolymer-Treated Soils

Suction measurement methods include the filter paper method [29], steam balance method (saturated salt solution) [29], pressure plate method [30], dewpoint water potential meter [31], etc. Specific test conditions and suction measurement ranges are shown in Table 2.

The principle of the filter paper method for measuring suction force in soil is that water vapor is exchanged between the filter paper and the soil sample in a closed space [30]. When the filter paper in the closed container fully exchanges the water or water vapor in the unsaturated sample to reach the equilibrium state, the free energy of the water in the sample can indirectly show the suction force of the soil, and when the filter paper and the water in the unsaturated sample exchange to the equilibrium state, their free energy state is the same. At this time, according to the rate curve in the filter paper method, the free energy of the water in the balanced filter paper is calculated and converted into the corresponding suction value—that is, the suction in the soil mass.

The Kelvin equation [29] is usually used to express this relationship:

$$s=-\frac{RT}{v_{w}}\ln(RH)$$

where *R* is the universal gas constant (J/(mol-K)), *T* is the absolute temperature (K), *ν_w_* is the molar volume of water, and *RH* is the relative humidity.

The vapor balance method involves placing the saturated salt solution in a closed glass container. Because different salt solutions have different physical and chemical properties, different humidity environments in the closed glass container can be controlled [30]. In the process of the vapor balance test, the water vapor exchange between the sample and the salt solution is carried out in a closed container to achieve balance, so that the suction value of different samples in the soil is controlled to reach the same humidity as that in the air—that is, the unified suction value. The corresponding suction value of the salt solution is shown in Table 3.

### 3.2. Microscopic Mechanism of Soil Water Retention Improvement by Biopolymers

**Figure 3:**
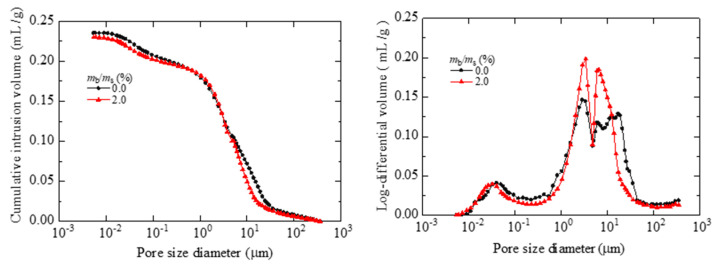
Mercury injection test results [32].

**Figure 4:**
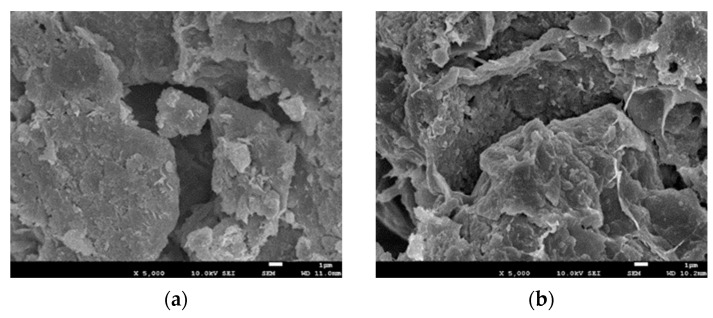

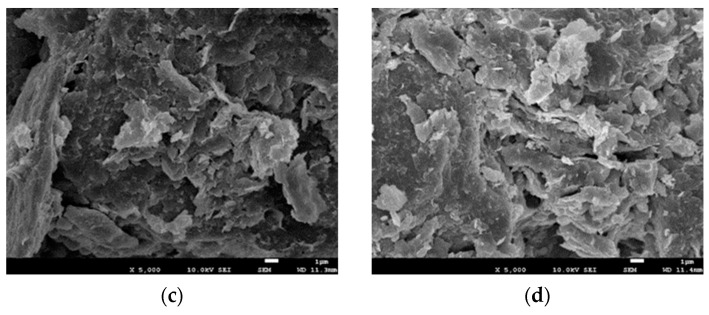
SEM images of silt treated with different biopolymers: (**a**) untreated silt, (**b**) xanthan-gum-treated silt, (**c**) gellan-gum-treated silt, and (**d**) guar-gum-treated silt.

The water retention properties of soils modified by biopolymers have been improved, and the mechanism of the improved water retention properties can be described from the microscopic perspective. Therefore, many researchers have begun to study the changes in soil pores from the microscopic perspective. The commonly used methods in geotechnical engineering include mercury injection [32], scanning electron microscopy [33], nuclear magnetic resonance [34], and so on. As shown in Figure 3 and Figure 4, the scanning electron microscope results of the above microscopic tests are more intuitive, and the mercury injection test results can quantify the pore changes in biopolymer-treated soil. According to the arrangement and distribution of polymers and soil particles in the soil, the polymer block adhesion within and between aggregates is defined as the cementation phenomenon formed due to bond cooperation [35]. The filling effect of the biopolymer on the pores between soil particles and the bonding of the biopolymer ions with charged soil particles can slow down the evaporation of water in the soil mass, thus improving the water-holding characteristics of the improved soil mass. In addition, under the action of multiple dry–wet cycles, changes in the microscopic pore structure of soil samples modified by biopolymers will become obvious [33]. Research on the micro scale is conducive to more convenient and intuitive characterization and interpretation of soil’s pore structure distribution and, furthermore, provides a more accurate and intuitive research method and means for the analysis of soil’s microstructure.

Moreover, compared with the results of several recent studies, it was found that the water content and pore ratio of the soils modified with biopolymers increased significantly after saturation. The water-holding capacity of the soils modified with xanthan gum, gellan gum, guar gum, and AGAR gum increased with the increase in the ratio, and the improvement effect of gellan gum was better than that of xanthan gum and guar gum. The mechanism is that, compared with untreated soil, biopolymer particles form hydrogels through hydration, fill the interparticle pores, and increase the adhesion between particles. Moreover, a pore space similar to a honeycomb structure is formed in silts modified with xanthan gum and gellan gum, providing space for water storage and ameliorating the water retention characteristics of the treated soil.

## 4. Mechanical Properties of Biopolymer-Treated Soils

Many studies have confirmed that biopolymers can improve the shear strength of soils, mainly in terms of the cohesion and the friction angle. Chang et al. [36] added 0.5–5% gellan gum to the sand, and the peak shear strength, cohesion (c), and friction angle (φ) were also significantly improved, due to the void-filling effect of gellan gum [37]. Biopolymers provide interparticle cohesion, increase strength through cohesion, and eventually bind the particles into clumps through interparticle connections. Cabalar et al. [38] pointed out that the strength of solidified sand was significantly increased with the increase in the xanthan gum ratio, and the compactness, viscosity, and strength of clay samples modified with xanthan gum were enhanced under low suction [39]. Further details of the use of various biopolymers to improve the shear strength, cohesion, and friction angle of different soils can be confirmed by Chang et al. [36] and Zhang et al. [3].

There are many factors affecting the strength of soil improved by biopolymers, such as moisture content, ambient temperature, type and proportion of biopolymer, and dehygroscopic path. Sujatha et al. [40] pointed out that the strength of the modified silt sample after curing and dewatering was higher than that before curing, indicating that water content is an important factor affecting the strength of the sample, which is also the significance of studying the changes in the strength characteristics of the improved soil while paying attention to the water retention characteristics. In addition, the strength of the sample is also related to the time and temperature of the specimen’s curing. Soldo et al. [41] pointed out that the shear strength of xanthan-solidified silt after curing can be significantly improved. From the perspective of the types of biopolymers, a variety of biopolymers have obvious improvement effects on the strength characteristics of different soils. Guar gum is more favorable for the treatment of collapsible soil and cohesive soil by wet mixing, while xanthan gum is superior in the treatment of silty fine soil [42,43]. In engineering practice, the dry and wet cycling caused by rainfall and evaporation will cause the strength attenuation of biopolymer-treated soils [44]. Nevertheless, the strength of soil modified with biopolymers is still higher than that of plain soil, and the residual strength of soil modified with biopolymers after multiple dry and wet cycles is still considerable [5]. Compared with sandy soil, the strength loss of clay is more significant [45]. At present, the research on the mechanical properties of soil improved by biopolymers mainly focuses on shear strength and tensile strength. The commonly used strength tests for soil improvement and reinforcement with biopolymers are: the direct shear test, unconfined tensile strength test, split test combined with the PIV test system, and triaxial test. Based on the conclusions of recent studies, several factors affecting the mechanical properties of biopolymer-treated soils were analyzed.

### 4.1. Effects of Types and Ratios of Biopolymers on the Mechanical Properties of Treated Soils

From the perspective of types, xanthan gum, gellan gum, guar gum, starch, chitosan, alginate, AGAR gum, pectin, polyurethane, and dextran can all improve the strength of soil [46]. The types of soil involved in this research include sand, silt, clay, fine sand, expansive soil, loess, etc. Guar gum and xanthan gum have the most significant influence on the mechanical properties of soil [47]. Ayeldeen et al. [42] found that guar gum was more suitable for the treatment of collapsibility and clay, while xanthan gum was superior for the treatment of silty fine-grained soil. For silt, xanthan gum and guar gum improved the unconfined compressive strength to 337 kPa and 842 kPa, respectively [48].

The molecular structure and polarity of biopolymers have different effects on the mechanical properties of different soils. Judge et al. [49] treated soft clay with four biopolymers of different polarity and structure (xanthan gum, guar gum, carrageenan, and dextran), and the test results of the neutral biopolymers guar gum and carrageenan were fundamentally different from those of the anionic xanthan gum and cationic dextran, while anionic biopolymers could better enhance the strength of the soil. The increase in the biopolymer ratio will significantly increase the cohesion of the soil and cause a reduction in the internal friction angle of the soil to improve its strength, and there is an optimal soil–biopolymer ratio, as has been demonstrated by some recent studies. For example, Chang et al. [7] reinforced sand with gellan gum, and both its shear strength and cohesion increased significantly with the increase in the ratio of the two biopolymers. Bagheri et al. [50,51] found that the unconfined compressive strength of soil improved with xanthan gum increased with the increase in the biopolymer ratio. Cabalar et al. [39] found that the strength of clay samples treated with xanthan gum (ratios of 0, 0.5, 1.0, 1.5, 2.0, and 3.0%) increased with the increase in the xanthan gum content. Fatehi et al. [52] also studied the effect of the biopolymer ratio on soil strength, using sodium alginate and AGAR to improve a kaolinite–sand mixture, and found that the optimal biopolymer content was in the range of 0.5–1% (relative to the soil weight). Gu et al. [53] concluded through direct shear tests on cellulose-improved soil that with the increase in the cellulose content, the shear strength of the soil first increased and then decreased, reaching a peak when the cellulose content was 0.5%.

Regarding splitting and brittle failure, Jiang et al. [32] concluded that different initial dry densities and xanthan gum contents had different effects on the mechanical properties of loess treated with xanthan gum. With the increase in the xanthan gum content, the strain softening phenomenon was more obvious, and the splitting tensile strength and brittleness of the sample increased.

### 4.2. Effects of Dry and Wet Cycling on the Mechanical Properties of Biopolymer-Treated Soils

**Figure 5:**
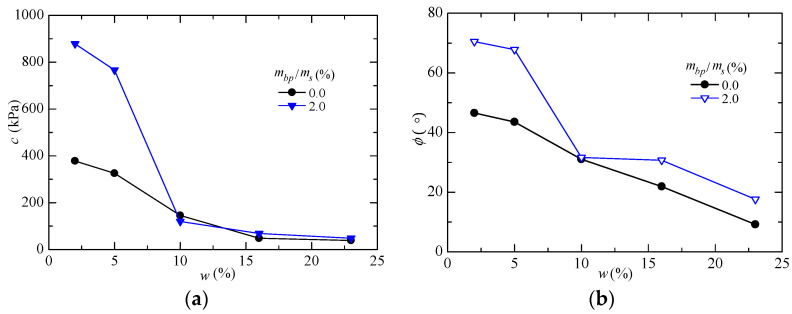
Changes in the c and φ values of silt treated with different ratios of xanthan gum: (**a**) Change in the c value of xanthan-gum-treated silt. (**b**) Change in the φ value of xanthan-gum-treated silt [3].

In the natural environment, soils improved with biopolymers will experience the cyclic process of rainfall infiltration and evaporation, which will affect the mechanical properties of the improved soil. The mechanical properties of soils modified by biopolymers will change during their transition from an unsaturated state to a saturated state. The results of several recent studies have proven that the strength of biopolymer-treated soil will decrease significantly with the increase in the water content and the number of dry and wet cycles. Figure 5 shows the changes in the c and φ values of xanthan-gum-treated silt under different ratios.

Chen et al. [54] found that the dry strength of xanthan-gum-improved sand was higher than its wet strength. Zhang et al. [3] found that the shear strength of xanthan-gum-treated silt decreased with the increase in the water content, and the cohesion and internal friction angle of the soil significantly decreased. Bagheri et al. [50,51] conducted an unconfined compressive strength test, triaxial test, and durability test under wet and dry cycling and the moisture sensitivity of soil improved with xanthan gum, and they found that the strength of soil improved with xanthan gum showed a decreasing trend after multiple wet and dry cycles. Chang et al. [5] found through experiments that the strength of Korean standard sand treated with gellan gum gradually decreased after dry and wet cycles, and the strength decreased by about 30% in 10 cycles. The results of several studies have shown that the strength attenuation after wet and dry cycling is due to the incomplete consistency between the decomposition and redrying of single-chain biopolymers during water absorption. Although the strength of treated soils will decrease under the action of dry and wet cycling, the study of Fatehi et al. [52] proved that biopolymers delayed the speed of soil strength reduction and the loss of soil particles, and the strength of treated soils was much higher than that of unimproved soils. Therefore, biopolymers still play a significant role in improving soil strength.

The specific effects of different dry and wet cycle conditions on various improved soils are the key directions for further research. Relevant research could better simulate the environmental characteristics of the engineering site, narrow the gap between the laboratory test and the engineering site, and make biopolymers that can be widely used in soil engineering.

### 4.3. Effects of Curing Age on the Mechanical Properties of Biopolymer-Treated Soils

Curing age refers to the time during which a stable structure is formed under certain environmental conditions after the soil is formed. Curing age is an important factor affecting the strength of improved soils [42]. The curing age generally ranges from 0 to 60 days, and the curing time plays a key role in improving the strength and deformation resistance of the improved soil [55]. In this period, the differences of various improved soils are relatively significant, and there is an optimal curing age. A recent related study found that the curing age at which an AGAR-modified sand–kaolin mixture reached its peak strength was 14 days [52]. In general, the strength indices of improved soil tend to increase with the increase in the curing age. Cabalar et al. [39,56] prepared samples by mixing different weight ratios of clay–xanthan gum biopolymers (0, 0.5, 1.0, 1.5, 2.0, and 3.0%) and then conducted tests at the end of different curing times (0, 7, 28, and 56 days), including unbounded compressive strength and shear tests. It was found that the strength of modified clay samples increased with the increase in the biopolymer content and curing time. Hataf et al. [57] studied the changes in the strength of chitosan-modified clay with curing age, and they believed that the nature of this change was mainly the changes in the soil water content and the structure of chitosan. Under wet conditions, the chitosan solution provides additional interactions between particles in the early stages; this effect attenuates over time, and the adhesion strength of the chitosan after drying is low.

**Figure 6:**
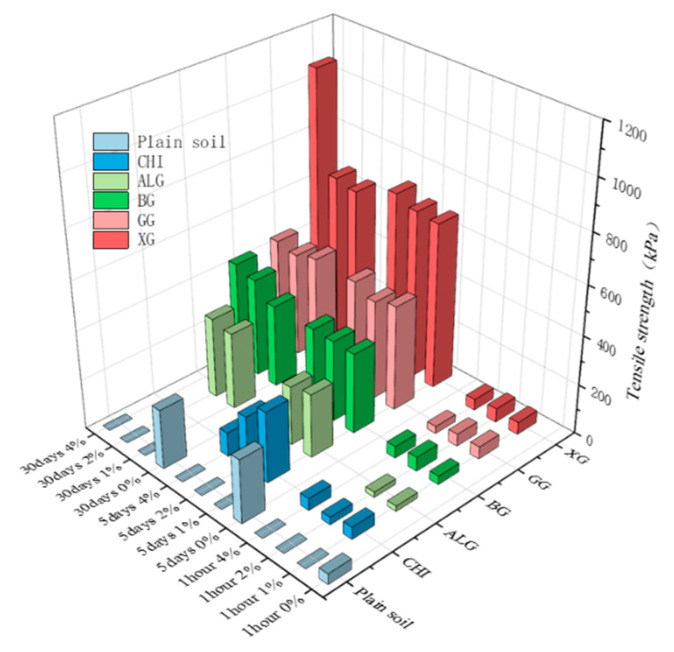
Changes in tensile strength under the influence of time, biopolymer type, and biopolymer concentrations; data cited from [46].

There is also an upper limit on the effect of the curing age, and the soil strength does not change significantly after a certain biopolymer concentration and curing time. Soldo et al. [41,46] studied five biopolymers: xanthan gum, β-1,3/1, 6-glucan, guar gum, chitosan, and alginate. The effects of unconfined compressive strength, splitting tensile strength, triaxial, and direct shear tests on the soil strength were studied. All of the tests were performed at different biopolymer concentrations and curing times. The splitting tensile results are shown in Figure 6. In the case of the unconfined compression, most of the strength was achieved during the first five days [46].

At present, the environmental problems are becoming more and more serious, and biopolymers are also used to treat municipal solid waste particles. Verma et al. [58] studied the treatment of municipal solid waste fine particles (MSWF) with xanthan gum and AGAR, cured at constant humidity and room temperature for 7–180 days. The results of triaxial tests showed that xanthan gum showed a slower strength growth rate over a period of up to three months, while MSWF treated with AGAR gum reached its final strength faster. AGAR shows better strength properties due to its better gel hardness. The long-term stability of biopolymer-treated MSWF offers a green approach that emphasizes circulability, sustainability, and the potential for energy savings and emission reductions.

### 4.4. Effects of Temperature Changes on the Strength of Biopolymer-Treated Soils

Based on the geographical location of the project site and the differences in ambient temperature caused by climate change throughout the year, the effect of temperature should be considered when applying biopolymer-treated soils to a specific project site. Moreover, there are many kinds of biopolymers, and the strength of soils modified by different biopolymers varies with temperature.

Many recent studies have considered the influence of temperature on the strength of biopolymer-treated soils. Bai et al. [59] found that the uniaxial compressive strength (UCS), secant modulus (E50), and tensile strength (TS) of different polyurethane-treated soils all increase linearly with the increase in temperature. Both positive and negative temperatures (i.e., 35 °C and 50 °C, −20 °C and −10 °C) have a positive effect on PU treatment. Positive temperature significantly improves the strength and ductility, while negative temperature significantly affects the strength and modulus. The linear relationships of E50-UCS and UCS-TS were obtained, which are closely related to temperature. Lemboye et al. [55] studied the effects of sodium alginate and pectin on the unconfined compressive strength (UCS) of sand. The UCS test measured the effects of the biopolymer type and concentration, curing interval and temperature, and water loss. The structural changes caused by the addition of the biopolymer were studied in combination with scanning electron microscopy (SEM). Curing temperatures between 25 and 110 °C affected the strength of acacia-treated sand samples. Bond decomposition occurs in sodium-alginate- and pectin-modified sand at temperatures higher than 110 °C, while bonds in acacia-modified sand remain stable at higher temperatures. Too high or too low ambient temperature will directly affect the mechanical properties of biopolymer-treated soils.

### 4.5. Analysis of the Dynamic Characteristics of Soils Treated with Biopolymers

Dynamic strength testing is an effective method to provide dynamic parameters for the seismic stability design of soil. Through the fatigue loading test, Ni et al. [60] proved that the addition of xanthan gum can effectively improve the fatigue life of clay under repeated loading. Im et al. [61] conducted resonance column tests on xanthan-gum-treated sandy soil, and the results showed that the addition of xanthan gum could effectively prevent the liquefaction of sandy soil.

There have been few relevant studies on the dynamic strength and dynamic deformation of biopolymer-treated soils, so it is necessary to study the dynamic deformation characteristics of biopolymer-treated soils based on unsaturated soil theory and soil–water characteristic curves, and to predict the dynamic deformation characteristics.

## 5. Permeability and Durability of Biopolymer-Treated Soils

Biopolymers can form fiber networks that inhibit water flow, so the soil permeability coefficient can be reduced by adding biopolymers. The durability of soils modified by biopolymers can be reflected by the erosion resistance, disintegration resistance, and the changes in the dry and wet cycling characteristics. Chang et al. [4] used wet construction to improve the soil mass of pedestrian walkways and found that the pedestrian walkways improved by biopolymer had high surface erosion resistance. Ng et al. [62] studied the effects of biopolymers on the gas permeability of compacted clay under different soil densities and compacted water contents, and they found that the gas permeability of modified clay containing biopolymers was always lower than that of pure clay, with a reduction of up to two orders of magnitude. Ham et al. [63] measured the surface erosion rate of biopolymer-treated sand at different water velocities and found that the surface erosion resistance and erosion resistance of dextran-improved saturated silt were significantly enhanced. Kumar et al. [64] found that compared with guar gum and β-glucan, xanthan gum can achieve a better osmotic reduction effect. In addition, in the presence of water, biopolymers expand into viscous hydrogels that fill pore spaces, resulting in reduced fluid permeability even in loose soils [40].

At present, most of the studies on the changes in soil’s permeability coefficient after biopolymer improvement have been conducted in the saturated state, but there have been few studies on the changes in the permeability coefficient in the unsaturated state. The permeability coefficient of soils treated with biopolymers will change during the transition from an unsaturated state to a saturated state. Modification of biopolymers can improve their effects. Trambitski et al. [65] studied the effects of a modified biopolymer (degraded corn starch) on the mechanical properties of clay. The technology for the preparation of a modified biopolymer was also introduced. The addition of modified polymers has significant effects on the properties and durability of clay materials. Incorporation of the modified polymer into the clay matrix contributed to a 62% increase in compressive strength (6.8 to 11 MPa). The durability of the clay materials was also significantly improved. In addition, the modified polymer also had a significant effect on the structure of the clay. Ni et al. [60] found that, compared with untreated soil, the xanthan gum–soil matrix can effectively improve the fatigue life or bearing capacity under repeated loading. While untreated soil failed at only half the stress level of UCS, xanthan-treated soil with 3.0% xanthan gum remained unchanged at the end of the trial. These data hint at the potential use of xanthan gum for soil stabilization under repeated loads.

## 6. Prediction Theory Related to Soil Properties Improved by Biopolymers

According to the unsaturated soil theory, suction is an important and key factor affecting the behavior of unsaturated soil, and the water retention properties, mechanical properties, and permeability properties of unsaturated soil are closely related to it. Many achievements have been made in the study of the water retention characteristics of unsaturated soils. A large number of experimental studies have shown that there are many factors affecting the water retention characteristics of soils, such as the initial pore ratio [66] and net vertical pressure [67].

Van Genuchten [68] and Fredlund and Xing [69] proposed the classical V-G model and the Fredlund–Xing model, respectively. The Fredlund–Xing equation is as follows:

$$w(s)=C(s)\frac{w_{s}}{\ln{\left[\exp(1)+{\left(s/a\right)}^{n}\right]}^{m}}$$

where *w(s)* is the gravimetric water content (%), *s* is the suction value (kPa), *S_re_* is the residual suction, *w_s_* is the saturated water content, *m* is a soil property parameter associated with the residual water content, *n* is a soil property parameter related to the slope of the inflexion point out of the SWRC, and *a* is a soil property parameter associated with the air-entry value. In addition, *C(s)* is a correction factor that allows the SWRC to pass through the point corresponding to a suction value of 106 kPa at 0% water content.

Zhou et al. [70] took the parameters in the Fredlund–Xing model as a function of the initial porosity ratio according to the test results and established their respective soil water retention characteristic prediction models. Zhang et al. [71] provided a semi-empirical formula for predicting the water retention properties of biopolymer-treated soils, which is based on the Fredlund–Xing model.

Many scholars have conducted a lot of research on the unsaturated soil strength model. Fredlund et al. [72] used saturation or effective saturation to consider the effective stress coefficient of the strength increase term caused by suction in the unsaturated soil strength formula. Alonso et al. [73] deducted the micropore saturation to consider the strength of unsaturated soil. Gao et al. [74] conducted experimental studies on the strength characteristics of unsaturated expansive soil and unsaturated silt within a wide suction range and proposed their respective prediction models. Zhou et al. [75] proposed a shear strength model of unsaturated soil based on capillary action. Lee et al. [76] found that three kinds of fine-grained soils had a series of different properties. The effects of interparticle forces on water content, particle size, particle separation, surface charge density, and the internal porosity of particles are complex functional relationships.

Recently, there has been a lot of work on the permeability characteristics of unsaturated soil. For example, direct methods have been used to study the permeability characteristics of unsaturated soil, including the steady-state method [77], transient profile method [78], and wetting front advance method [79]. For example, the unsaturated soil permeability coefficient model proposed by Van Genuchten [68] and Fredlund et al. [69], based on the soil–water characteristic curve, has been widely used in indirect research. Cai et al. [80] put forward a prediction model for the unsaturated soil permeability coefficient that can take into account the change in the pore ratio. Gao et al. [66] proposed a prediction model for the unsaturated soil permeability coefficient that could take into account pore ratio changes and hysteresis effects.

However, there are few predictions about the permeability properties of biopolymer-treated soils, which will be the key to further study.

## 7. Biopolymer-Treated Soil Engineering Field Implementation

The application of biopolymers in the treatment of foundation soil at engineering sites is the future goal of related research. Hamza et al. [81] introduced the test results of highly plastic soil reinforced with xanthan gum (XG) biopolymer to determine whether it was suitable for use as road subgrade. The XG ratios ranged from 0% to 5%, and the samples were tested at different aging periods (0–60 days). The results showed that XG biopolymers have good potential as an alternative admixture for the treatment of widely distributed high-fat subgrade soil, which can transform weak subgrade into hard subgrade for pavement construction.

## 8. Conclusions and Future Prospects

In terms of water retention characteristics, most of the current studies have focused on the water retention characteristics of biopolymer-treated soils in the low suction range, while few studies have considered the hysteresis effect of the dehygroscopic curve in the full suction range. When the modified soil changes from an unsaturated state to a saturated state, the water retention characteristics of the biopolymer-modified soil will change, and it is necessary to pay more attention to this change process.

In terms of mechanical properties, there have been more studies on the mechanical properties of biopolymer-treated soils under the optimal moisture content state, but few quantitative studies on the mechanical properties of biopolymer-treated soils under the full suction range, and also few quantitative studies on the mechanical properties of biopolymer-modified soils under the dry–wet cycle controlled by suction. When the biopolymer-modified soil changes from an unsaturated state to a saturated state, the mechanical properties of the biopolymer-modified soil will change. It is necessary to quantitatively study the mechanical properties of biopolymer-treated unsaturated soil in the full suction range.

In terms of permeability characteristics, the research on the permeability coefficients of soils modified with biopolymers is mostly in the saturated state, while the changes in the permeability coefficients in the unsaturated state are rarely studied. The permeability coefficients of soils modified with biopolymers will change during the transition from an unsaturated state to a saturated state.

In summary, soils modified with biopolymers have higher strength and stronger water-holding capacity, and they also show a certain durability. Continuing to expand the research results of biopolymer-treated soils based on unsaturated soil theory, there will be an optimal method for the improvement of the soil engineering properties of biopolymers. The preparation method of modified biopolymers and their effects on soil improvement need to be further studied to adapt to various engineering sites and soil types.

## Figures and Tables

**Table 1:** Summary of commonly used biopolymers in geotechnical engineering.

Biopolymers	Action Effect	Advantage	Defect	References
AGAR	Increased strength Porosity reduction Resistance to erosion	Improves strength and durability	Serious pollution	[4,6,7,8]
Gellan gum	Increased strength Improved water retention	Better water retention	Needs a high-temperature environment	[5,9]
Guar gum	Blocking pores Dust reduction Increased strength Grouting	High viscosity Strong hydration	Higher cost	[10,11]
Xanthan gum	Increased strength Improved water retention	Low cost	-	[12,13]
Chitosan	Reduced heavy metals	Reduced water pollution	High cost	[14,15,16,17,18]
Starch	Increased strength Resistance to erosion	Easy to obtain	High cost	[19,20,21,22,23]

**Table 2:** Test method for the water retention properties of biopolymer-treated soil.

Suction	Suction Control Method	Specific Suction Range	Specimens’ Size/mm	Biopolymer Ratio Range (m_bp_/m_s_)
Low suction	Pressure plate	0–1500 kPa	*d* = 61.8, *h* = 20	0.5~3%
High suction	WP4C	0–300 MPa	*d* = 33.0, *h* = 7	0.5~2%
High suction	Filter paper	0.2–100 MPa	*d* = 61.8, *h* = 20	0.5~3%
High suction	Steam balance	3–370 MPa	*d* = 61.8, *h* = 20	0.5~2%

**Table 3:** Saturated salt solution and corresponding suction value (20 °C).

Saturated Salt Solution	Relative Humidity/%	Total Suction/MPa
LiBr	6.6	367.54
LiCl·H_2_O	12.0	286.70
CH_3_COOK	23.1	198.14
MgCl_2_·6H_2_O	33.1	149.51
K_2_CO_3_	43.2	113.50
NaBr	59.1	71.12
KI	69.9	48.42
NaCl	75.5	38.00
KCL	85.1	21.82
K_2_SO_4_	97.6	3.29

## Data Availability

The data presented in this study are available upon request from the corresponding author.
